# Editorial: Oxytocin and Social Behaviour in Dogs and Other (Self-)Domesticated Species: Methodological Caveats and Promising Perspectives

**DOI:** 10.3389/fpsyg.2019.00732

**Published:** 2019-04-05

**Authors:** Anna Kis, Jessica Lee Oliva, Zsófia Virányi, József Topál

**Affiliations:** ^1^Institute of Cognitive Neuroscience and Psychology, Hungarian Academy of Sciences, Budapest, Hungary; ^2^Faculty of Medicine, Nursing and Health Sciences, School of Psychological Sciences, Monash University, Clayton, VIC, Australia; ^3^Comparative Cognition, Messerli Research Institute, University of Veterinary Medicine, Vienna, Austria; ^4^Medical University of Vienna, University of Vienna, Vienna, Austria

**Keywords:** domestic species, oxytocin, social behavior, dog, intranasal administration, gene-behavior associations

Over the past decade the oxytocin system has become a focus of attention for researchers from various fields studying mechanisms underlying different forms of social behavior. Some have even suggested that it is the neurohormone, oxytocin, that has had the most permissive role in the evolution of the human nervous system (Carter, 2014), implying that *Homo sapiens* could not have evolved without it, as the success of this species highly depends on social behavior and cognition. Not surprisingly research into model systems of human social behavior has followed this trend including several discoveries on the relatedness of numerous forms of domestic species' social behavior and their respective oxytocin systems. This is particularly interesting as domestic species are known to have adapted to the human social environment in evolutionary terms, however the proximal and distal mechanisms underlying behavioral parallels between humans and domestic animals still remain largely unexplored.

Among domestic species, dogs are the most studied model of human behavior, and their human-analog socio-cognitive skills have been well-established both at the behavioral (Miklósi and Topál, 2013) and at the neural (Bunford et al., 2017) level. This bias in favor of dogs is also present in the number of research papers published about oxytocin and social behavior, as considerable amount of information has already accumulated about this species over the past few years (reviewed in Kis et al., 2017). The aim of this special issue was to fill in gaps not only for canine oxytocin research, but also for research on other domestic species. An important critical review article by Rault et al. presents literature on dogs, pigs, cattle, and sheep focusing on welfare aspects and outlines both problems and possible solutions for oxytocin research. The other 16 articles in the special issue present original research that keep up with the high methodological standards and present valuable data that the field had thus far been missing.

These include, first of all, research on non-canine domestic species. Bienboire-Frosini et al. present a reliable immunoassay measure of peripheral (plasma) oxytocin in cat, dog, horse, cow, pig, sheep, and goat. Arahori et al. focus on domestic cats and describe microsatellite polymorphisms adjacent to the oxytocin receptor gene revealing moderate associations with owner-rated personality traits.

The remaining 14 original research papers present data on domestic dogs' oxytocin system and social behavior. These include research using various methodological approaches: gene × behavior associations, intranasal oxytocin administration, and peripheral oxytocin measurements. Among the genetic studies is one (Cimarelli et al.) that presents a considerable methodological advance in the field, introducing an epigenetic study and presenting, for the first time, evidence that oxytocin receptor gene (OXTR) methylation is associated with social behaviors of pet dogs. A similarly important conceptual novelty is the introduction of quantitatively measured environmental factors (OXTR polymorphisms in the owners' gene; Kovács et al.) as well as contextual and individual characteristics (Turcsán et al.) into canine OXTR research. Both of these studies highlight significant additional factors to genetic research into dogs' oxytocin system, although at present have only tested these on one specific breed, the Border collie. The importance of breed differences, on the other hand, is highlighted by another paper (Kubinyi et al.) that presents incremental research about the relationship between OXTR polymorphisms and greeting behavior. Building on their previous research on Border collies and German shepherds the authors show a similar relationship in Siberian huskies. Direct comparison with human subjects (infants) is carried out by Oláh et al. investigating gaze-following as a function of OXTR polymorphisms.

The special issue also includes significant new research using intranasal administration of oxytocin (IN-OT) for dogs. While the literature of the field seems to be biased toward reporting of positive results, an important negative finding is highlighted here by Thielke et al., in a study investigating the effects of oxytocin administration on dogs' attachment behavior toward their owners measured via the strange situation test. Results show that contrary to expectations, intranasal administration of oxytocin fails to increase owner-directed proximity and contact seeking, rather it decreases such behaviors (in the baseline phase). A very interesting pair of papers by Somppi et al. and Kis et al. used eye-tracking technology to assess how oxytocin administration modulates dogs' viewing of human faces with different emotional expressions. The two research groups independently carried out studies using the same set of stimuli, and while the general conclusion from both is that there is an effect of oxytocin on the outcome measure, the specifics of the results differ by several points. The special issue also includes a paper presenting important incremental research using IN-OT methodology (Nagasawa et al.), which shows that previous results of the same research group about enhanced gazing behavior following oxytocin treatment can be conceptually replicated in ancient Japanese breeds (Shiba, Kai, and Shikoku).

Studies about canine peripheral oxytocin levels in this special issue include methodological improvements as well as the assessment of different forms of dog-human interaction. Temesi et al. describe the time-course of intranasally and intravenously administered oxytocin on serum and urine oxytocin concentrations by directly comparing these measures in Beagle dogs. MacLean et al. validate their salivary oxytocin measure by comparing it to plasma measures of the same Labrador retriever and Labrador retriever × Golden retriever dogs before and after a free-form social interaction with a human versus control treatment (resting). Two of the papers (Petersson et al.; Rossi et al.) measure both oxytocin and cortisol levels from dogs' blood samples and find that the two hormones are related to behavior during their respective tests. Another paper by MacLean et al. connects to the applied value of oxytocin research focusing on dogs' aggressive behavior: while dogs with and without reported history of aggression only differ in plasma vasopressin levels (and not oxytocin), an interesting difference is found between pet dogs and assistance dogs (that have been bred for affiliative and non-aggressive temperaments), with the latter group having higher oxytocin levels.

In our view the papers of this special issue present a considerable advancement in the field of oxytocin research in domestic species. While the independent research papers collected here use varying methodology and address independent scientific questions, they all nicely tie to the conceptual and methodological gaps that have been highlighted. Studies including non-canine domestic species, as well as different breeds of dogs inform us about both the specificity and the generalizability of oxytocin effects. Conceptual replications and incremental research presented here ensure the robustness of the findings. The novel methodological approaches as well as conceptual innovations described in this issue broaden the scope of the field. Furthermore, the open reporting of negative and controversial findings guarantees transparency of research. The papers of this issue are all good examples for this, and thus together strengthen the view that domesticated animals serve as valuable models for investigating the interrelatedness of social behavior and the oxytocin system.

## Author Contributions

AK drafted the first version of the manuscript. All authors read and commented the manuscript as well as contributed to its content and approved of the final text.

### Conflict of Interest Statement

The authors declare that the research was conducted in the absence of any commercial or financial relationships that could be construed as a potential conflict of interest.
